# Angelica Yinzi alleviates 1-chloro-2,4-dinitrobenzene-induced atopic dermatitis by inhibiting activation of NLRP3 inflammasome and down-regulating the MAPKs/NF-kB signaling pathway

**DOI:** 10.1016/j.jsps.2022.07.003

**Published:** 2022-07-19

**Authors:** Wei Liu, Wanci Song, Yang Luo, Hanxiong Dan, Li Li, Zhouyang Zhang, Daonian Zhou, Pengtao You

**Affiliations:** a Beijing Key Laboratory of Molecular Pharmaceutics and New Drug Delivery Systems, School of Pharmaceutical Sciences, Peking University, Beijing 100191, PR China; b Hubei Key Laboratory of Resources and Chemistry of Chinese Medicine, Hubei University of Chinese Medicine, Wuhan, Hubei 430065, PR China; c Post-doctoral Research Center of Mayinglong Pharmaceutical Group Co., Ltd., Wuhan, Hubei 430060, PR China; d Department of Pharmacy, Wuhan Hospital of Traditional Chinese Medicine, Wuhan, Hubei 430014, PR China

**Keywords:** Angelica Yinzi, Atopic dermatitis, MAPK, NLRP3, Inflammation

## Abstract

**Background:**

Atopic dermatitis (AD), characterized by eczema as a chronic pruritic inflammatory skin disease, has become a serious health problem with recurrent clinical episodes. However, current clinical treatments have limited relief and are accompanied by adverse effects. Therefore, there is a necessity to develop new effective drugs for AD treatment. Angelica Yinzi (AYZ) is a classic ancient prescription for nourishing blood, moistening dryness, dispelling wind, and relieving itching. However, its mechanism for alleviating atopic dermatitis remains unknown. Therefore, this study aimed at determining the effects of AYZ and its potential mechanism in alleviating AD-like symptoms.

**Methods:**

In the present study, we used 1-chloro-2,4-dinitrobenzene (DNCB) to establish a mouse model of atopic dermatitis, where DNCB readily penetrates the epidermis to cause inflammation. Histopathological analysis was performed to examine the thickening of dorsal skin and infiltration in the inflammatory and mast cells in C57BL/6 mice. Additionally, the immunoglobulin E (IgE) levels in serum were determined by enzyme-linked immunosorbent assay (ELISA) kits. The IL-1β and TNF-α expression were detected using qRT-PCR. Next, the Western blotting and immunohistochemistry assays were performed to assess the contribution of MAPKs/NF-κB signaling pathways and the NLRP3 inflammasome in AD responses.

**Results:**

Histopathological examination revealed that AYZ reduced the epidermal thickness of AD-like lesioned skin and repressed the infiltration of mast cells into AD-like lesioned skin. AYZ significantly decreased the phosphorylation of p38 MAPK, JNK, ERK and NF-κB and downregulated serum IgE levels and IL-1β and TNF-α mRNA levels. Additionally, the NLRP3, ASC, Caspase-1, and IL-1β expression in dorsal skin were effectively down-regulated following AYZ treatment (*p* < 0.05 and *p* < 0.01).

**Conclusion:**

These findings revealed that AYZ effectively suppressed AD-induced skin inflammation by inhibiting the activation of the NLRP3 inflammasome and the MAPKs/NF-kB signaling. Therefore, AYZ is a potential therapeutic agent against AD in the clinical setting.

## 1 Introduction

Atopic dermatitis (AD) is a chronic, pruritic, recurring inflammatory disease of the skin, affecting 20% of children and 10% of adults in many high-income countries (Czarnowicki et al., 2019, Langan et al., 2020). Its primary symptoms include lichenification on dry skin and eczematous lesions linked to mental health problems, such as sleep disorders and fatigue. Damaged skin is characterized by elevated serum IgE levels and infiltration of inflammatory cells (lymphocytes, macrophages, eosinophils and mast cells) (Barton and Sidbury, 2015). For instance, the activation of mast cell infiltration contributes to AD and is easily noticed in skin tissue with AD (Gonzalez-de-Olano and Alvarez-Twose, 2018). In addition, the MAPKs phosphorylation induces the production of inflammatory mediators and allergic inflammatory responses (Huang et al., 2019, Park et al., 2019). For example, the NLRP3 inflammasome activated by the activation of the NF-κB pathway regulates contact allergy (Meng et al., 2009). Furthermore, the immune system produces high levels of proinflammatory mediators, including cytokines (IL-1β, TNF-α), which play an important role in host cell defense (Liu and Ding, 2019). Although AD is associated with immune system disorders, genetics, skin barrier disruption, and environmental factors, its diverse pathogenesis has not been clearly elucidated (Torres et al., 2019, Yan, et al., 2019).

Currently, AD is treated with topical corticosteroids, topical calcineurin inhibitors, and systemic immunotherapies (Newsom et al., 2020). However, these treatments have a rebound phenomenon, adverse side effects, and intermittent recurrencies (Hou et al., 2017). For example, the local cutaneous atrophy, striae, and stinging are side effects of the long-term use of topical calcineurin inhibitors (Lee et al., 2020, Paller et al., 2016). Therefore, safer and more effective AD treatments against AD, such as traditional Chinese medicine (TCM), have recently attracted increased and widespread interest (Eyerich and Novak, 2013).

The TCM has unique advantages in AD routine management and treatment. The efficacy of its classic herbal formulation AYZ is scientifically and clinically proven and has been widely used in the treatment of chronic urticaria, hypersensitivity, pruritus and AD without any severe adverse events (Qin et al., 2020). The AYZ formula comprises of 11 different herbs including, *Angelica sinensis (Oliv.) Diels*, *Paeonia lactiflora Pall.*, *Ligusticum chuanxiong Hort.*, *Rehmannia glutinosa Libosch., Tribulus terrestris L.*, *Saposhnikovia divaricate (Trucz.) Schischk.*, *Schizonepeta tenuisfolia Briq.*, *Polygonum multiflorum Thunb.*, *Astragalus membranaceus (Fisch.) Bge.*, *Glycyrrhiza uralensis Fisch.* and *Zingiber officinale Rosc*. Recent pharmaceutical studies have demonstrated that *A. sinensis (Oliv.) Diels* and *L. chuanxiong Hort.* have anti-inflammatory effects induced via the NF-κB and MAPKs signaling pathways (Gu et al., 2018, Lee et al., 2010). In addition, *P. lactiflora Pall.* and *S. tenuisfolia Briq.* have been used to reverse the effects of AD (Choi et al., 2013, Jo et al., 2018). A study has shown through a network pharmacology approach that AYZ may intervene in AD by acting on MAPKs/NF-kB signaling pathway(Wang, 2021). Therefore, based on the above studies, this study focused on establishing the underlying mechanism of AYZ in ameliorating DNCB-induced AD in mice.

## 2 Materials and methods

### 2.1 Reagents

AYZ was obtained from the Mayinglong Pharmaceutical Co., Ltd (China). ShiDuQingPian (SDQP) was purchased from Guangxi Yulin Pharmaceutical Group Co., Ltd (China). Cetirizine hydrochloride tablet (CHT) was purchased from Dong rui Pharmaceutical Co., Ltd (China). 1-chloro-2,4-dinitrobenzene (DNCB) and olive oil were purchased from Shanghai McLean Biochemical Technology Co., LTD.

### 2.2 Animals and AD induction

**Fig. 1:**
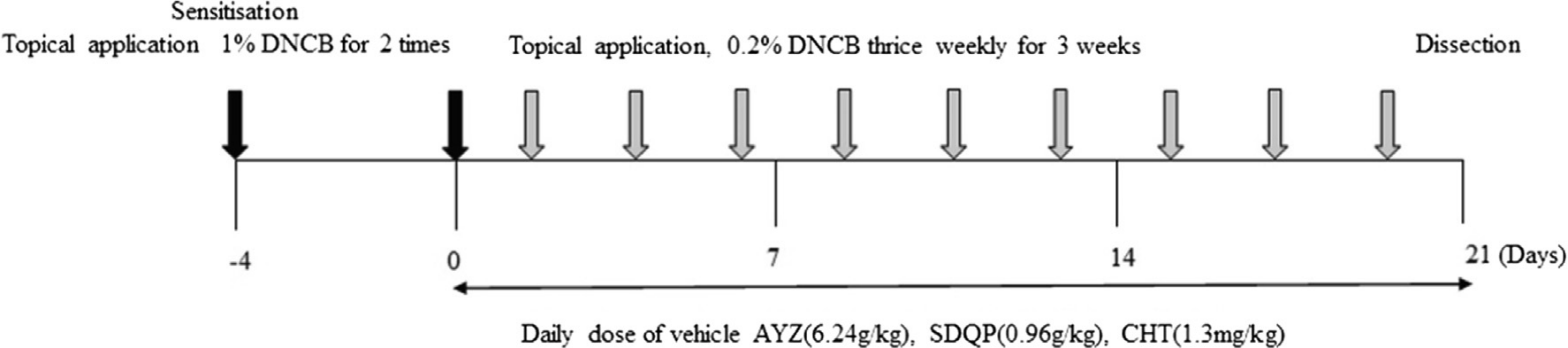
C57BL/6 mice were treated with 1% DNCB in acetone/olive oil (3:1) on days −4 and 0. Then, mice were challenged with 1% DNCB thrice weekly for three Then, mice were challenged with 1% DNCB thrice weekly for three weeks (days 1–21). AD-like lesions were treated with AYZ (6.24 g/kg) or SDQP (0.96 g/kg) or CHT (1.3 mg/kg) for three weeks.

Male C57BL/6 mice (6–8 weeks old) weighing 18–20 g were purchased from Hubei Provincial Center for Disease Control and Prevention (SCXK2017-0012). The mice were housed in individually-ventilated cages maintained at 22 ± 2 °C, the humidity of 50 ± 10%, and 12:12-h light: dark cycle and were provided with adequate food and water. Atopic dermatitis-like immunological and skin lesions were induced on the dorsal skin, face, and back of both ears on each mouse using DNCB. Specifically, approximately 3 cm^2^ patch was shaved on each mouse back using an electric clipper a day before DNCB treatment. The 75 mice were randomly assigned into five groups (n = 15 per group), namely the control, DNCB, DNCB plus oral AYZ, DNCB plus oral SDQP, DNCB plus oral CHT groups based on the treatments given. The experimental design is summarised in Fig. 1. For AD sensitization, 200 μl of 1% DNCB solution (dissolved in acetone and olive oil in the ratio 3:1) was applied repeatedly on the face and backs of both ears twice on days −4 and 0. To induce AD-like lesions, 200 μl of 0.5% DNCB solution was applied on the dorsal skin thrice weekly for three weeks (days 1–21). Upon sensitization, 20 ml/kg or 6.24 g/kg of AYZ was orally administered daily to the AYZ treated group for three weeks. The SDQP treated group (20 ml/kg, daily oral 0.96 g/kg) and CHT treated group (20 ml/kg, daily oral 1.3 mg/kg) served as the positive controls. The DNCB group was orally administered with the same volume of pure water. All operations were orderly carried out in accordance with the serial number of mice. The mice were sacrificed and samples were collected. Mice in the control group were treated with vehicles.

### 2.3 Evaluation of dermatitis severity

Dermatitis was scored weekly following the previously described criteria with slight modifications (Kim et al., 2014). Erythema/bleeding (I), edema (II), scratch/erosion (III) and scale/dryness (IV), were scored as 0 (none), 1 (mild), 2 (moderate) or 3 (severe). The total score of dermatitis ranged from 0 to 12. To minimize technical differences, a single investigator performed all the measurements across the experiment.

### 2.4 Histological analysis

The mice's skin tissues were fixed in 10% neutral buffered formalin (NBF) for 24 h then embedded in paraffin and sectioned into blocks with a 5 μm thickness. To evaluate the tissue architecture and the degree of mast cell infiltration, the tissue sections were stained with a hematoxylin and eosin (H&E) solution and toluidine blue (TB) (Servicebio). The Stained sections were visualized and images captured under a light microscope (Olympus, Japan).

### 2.5 Immunohistochemistry analyses

The paraffinized skin tissue was dewaxed, dehydrated then antigen retrieval was performed. Next, the non-specific binding was blocked by placing the tissues in 0.3% hydrogen peroxide for 15 min then in 5% bovine serum albumin (BSA) for 30 min. The expression of NLRP3, ASC, caspase-1, or p-NF-κB proteins were primarily labeled with NLRP3 antibody (1: 100 dilution; Abways, China) (CY5651), ASC antibody (1: 100 dilution; Abways, China) (AY3812), Caspase-1 antibody (1: 200 dilution; Bioss, China) (bs-0169R) or p-NF-κB antibody (1: 200 dilution; CST, USA) respectively overnight at 4 °C. The tissues were then incubated with the secondary horseradish peroxidase (HRP)-conjugated anti-rabbit IgG antibody (1: 500 dilution; DAKO, K5007) for 60 mins to localize the primary antibody binding. The immunohistochemistry assessment was performed using the DAB (3, 3′-diaminobenzidine) staining kit (Boster, Wuhan, China). The stained tissues were observed, and images were captured under an Olympus microscope.

### 2.6 Determination of the serum IgE levels

The mice blood samples were centrifuged at 2000 g at 4 °C for 20 min. The obtained serum was stored at −80 °C awaiting further analysis. The mouse IgE ELISA kits were purchased from Shanghai Fusheng Industrial Co., LTD. (A105159). The total serum levels of IgE were measured using mouse IgE ELISA kits according to the manufacturer’s instructions.

### 2.7 Western blotting

The dorsal skin proteins were analyzed by western blotting. First, the skin tissues were homogenized using a radioimmunoprecipitation assay buffer supplemented with protease inhibitors. Next, the protein concentration in each sample was detected using an enhanced BCA protein assay kit. The proteins were then denatured in sodium dodecyl sulphate (SDS) buffer, separated on a 10% SDS-polyacrylamide gel electrophoresis, and transferred onto the polyvinylidene difluoride (PVDF) membranes. The membranes were then blocked with 5% BSA at room temperature for 2 h, and incubated overnight at 4 °C. The membranes were then incubated with several primary antibodies, including GAPDH (1:1000 dilution, Cell Signaling Technology, #5174), P-ERK (1:1000 dilution, Cell Signaling Technology, #4695), P-JNK (1:1000 dilution, Cell Signaling Technology, #4668), P-P38 (1:1000 dilution, Cell Signaling Technology, #9910), P38 (1:1000 dilution, Cell Signaling Technology, #8690), NF-κB (1:1000 dilution, Cell Signaling Technology, #8242), and P-NF-κB (1:1000 dilution, Cell Signaling Technology, #3033) overnight at 4 °C. The membranes were rinsed in three changes of tris buffered saline + Tween (TBST) then incubated with the anti-rabbit secondary antibody (1:2000 dilution, Cell Signaling Technology, #4412) diluted in 5% non-fat milk at room temperature for 1 h. The membranes were then washed in three changes of TBST for 10 min. Proteins were visualized using ECL (Thermo), and membranes were scanned and imaged by the FluorChem FC3 system (ProteinSimple, USA).

### 2.8 Quantitative reverse transcription-polymerase chain reaction (qRT-PCR)

The total RNA was extracted from dorsal skin samples using the Trizol reagent (Thermo Fisher Scientific, USA). The spectrophotometric values of A260/280 ranged from 2.0 ∼ 2.4 and the values of A260/230 were 2.1–2.2, indicating that the isolated RNA was free of polyphenols, polysaccharides and protein contaminants. Next, 1 μg of total RNA was reverse transcribed into cDNA using HiScript® III-RT SuperMix (Vazyme biotechnology, Nanjing, China). The primers were designed using the Primer Premier 5.0 design software (Premier, Canada) and synthesized by Sangon Biotech (Shanghai, China). The mRNA expression levels of target genes were then determined using ChamQ Universal SYBR qPCR Master Mix (Vazyme) with the thermal cycling conditions: pre-denaturation at 95 °C for 30 s, denaturation at 95 °C for 10 s, denaturation at 60 °C for 30 s, and then melting curves were generated at 95 °C for 15 s, 60 °C for 60 s, and 95 °C for 15 s. The mRNA expression with GAPDH as an internal control was normalized. The relative quantification was performed using the 2-ΔΔCT method. The primers for *IL-1β* are as follows, Forward primer: 5′-CATCCAGCTTCAAATCTCGCAG-3′; reverse primer, 5′-CACACACCAGCAGGTTATCATC-3′. The primers for *TNF-α* are as follows, Forward primer: 5′-CATCTTCTCAAAATTCGAGTGACAA-3′; reverse primer, 5′-CATCTTCTCAAAATTCGAGTGACAA-3′. The primers for *GAPDH* are as follows, Forward primer: 5′-CATGGCCTTCCGTGTTCCTA-3′; reverse primer, 5′-CCTGCTTCACCACCTTCTTGAT-3′.

### 2.9 Chemical analysis of AYZ

The Chinese herbal formula AYZ was prepared from a TCM concoction consisting of 11 Chinese medicinal plants extracts. The chemical compounds present in AYZ were then determined using an UHPLCQ-TOF-MS consisting of a quaternionic pump (LC-20 AT), an array detector (DAD), electrospray ion source (ESI) At an ultimate UHPLC XB C18 spectrum of 2.1 × 100 mm, 1.8 μm, and wavelengths set at 230 nm. The column thermostat was maintained at 35 °C and the mobile phase comprised of 0.05% Formic acid acetonitrile mixture (A) and 0.05% formic acid (B). The elution gradient was as follows: 0–3.5 min, 5–15% A; 3.5–6.5 min, 15–26% A; 6.5–7.5 min, 26–27% A; 7.5–10 min, 27–40% A; 10–14.5 min, 40–90% A, and 14.5–17 min, 90–5% A. An injection volume of 1 μl, with a flow rate of 0.4 ml/min was used. The LC-MS data were collected by Agilent Mass Hunter (B.08.00) software and processed using the Agilent software, Qualitative Navigator (B.08.00), and Qualitative Workflows (B.08.00).

### 2.10 Statistical analysis

All quantitative data derived from this study were analyzed statistically. The results are expressed as the mean ± standard deviation (SD) of at least three separate tests. All data analyses were performed using the two-tail, equal variance Independent-Samples *t*-test and one-way ANOVA in the SPSS 22.0 software (IBM, USA). *p* < 0.05 were considered statistically significant.

## 3 Results

### 3.1 AYZ alleviated DNCB-induced AD-like symptoms

The DNCB group exhibited severe erythema, hemorrhage, erosion, and dryness compared to the control. However, after treatment with AYZ 3 weeks, the DNCB-induced AD severity was significantly decreased in the AYZ group (Fig. 1A). The dermatitis score was also significantly higher in the DNCB group than in the control group (*p* < 0.01; Fig. 1B). Similarly, AYZ treatment significantly decreased the dermatitis score (*p* < 0.01). The serum IgE levels were also increased in the DNCB group compared to the control group but were significantly reduced by AYZ treatment, suggesting that it suppresses the IgE synthesis associated with AD (*p* < 0.01; Fig. 1C). The repeated DNCB exposure also induced potent inflammatory changes, including the skin dermis and epidermis thickening in the DNCB group compared to the control group (Fig. 1D). Therefore, there were few mast cells in the AYZ group and positive groups 21 days after treatment. These findings imply that AYZ has an alleviatory effect against AD clinical symptoms and prevents severe AD pathological states.

### 3.2 AYZ regulated MAPKs and NF‐κB signaling pathways

**Fig. 2:**
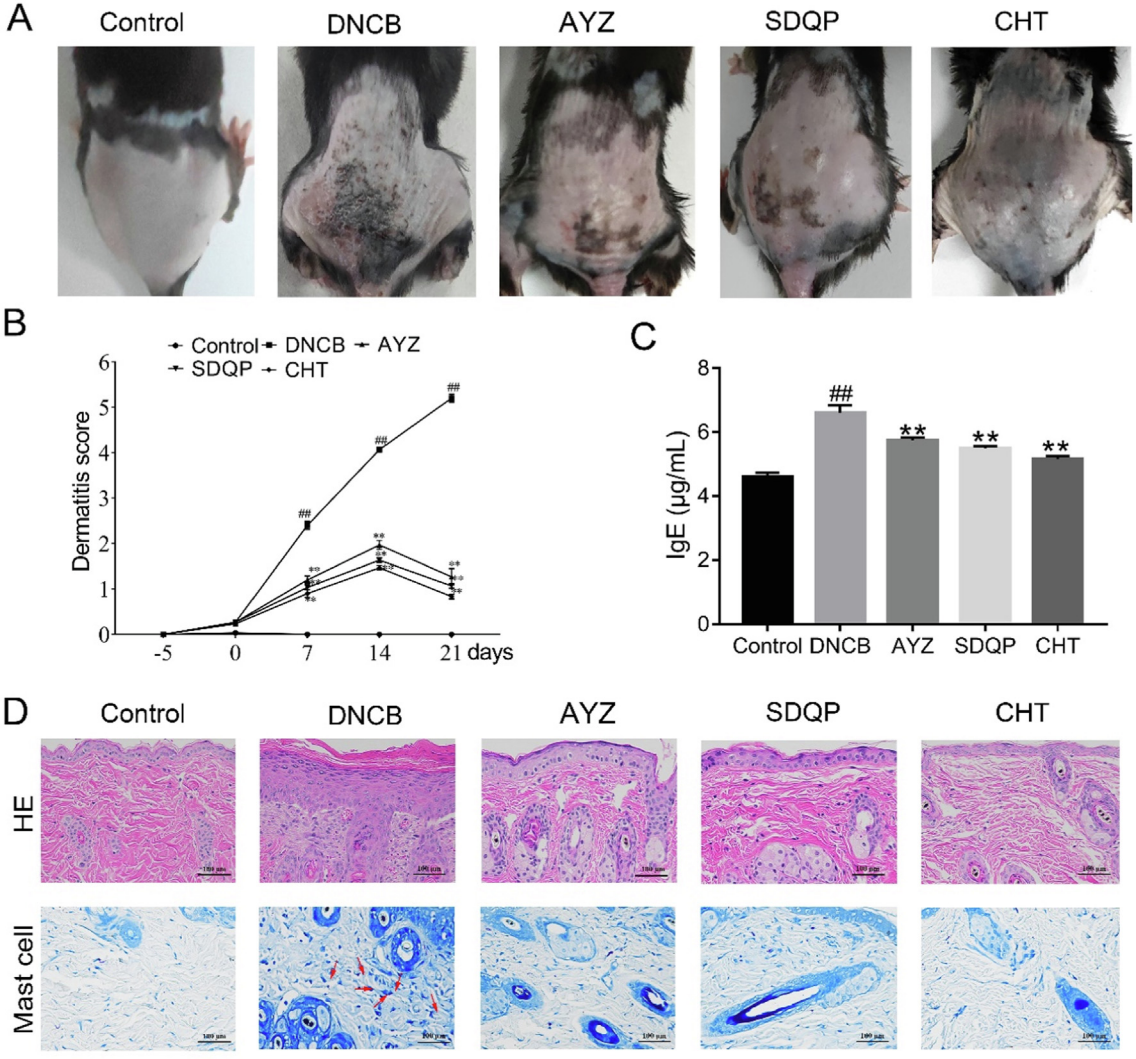
Angelica Yinzi attenuates DNCB-induced AD-like skin inflammation in C57BL/6 mice. (A) Clinical features in mice treated with DNCB, AYZ, SDQP, CHT, and the control (B) Dermatitis scores on DNCB, AYZ, SDQP, CHT, and the control groups. (C) Total IgE levels in serum of mice treated with DNCB, AYZ, SDQP, CHT, and the control. (D) Representative photomicrographs of skin sections stained with H&E and TB (×200). In the TB staining panel, red arrows denote mast cells. Data are expressed as the mean ± SD. (n = 3). ^##^*p* < 0.01, vs. the control group. *p < 0.05 and **p < 0.01 vs. the DNCB group.

The p-p38, p-ERK, p-JNK, p-p65 expressions were increased in the DNCB group relative to the control group (*p* < 0.01; Fig. 2A-B). However, AYZ alleviated the DNCB-induced increase in p-p38, p-p65, p-ERK, and p-JNK expressions relative to the DNCB group (*p* < 0.05 and *p* < 0.01). In addition, there were no significant differences in p65 expression between the experimental groups. The immunohistochemical staining to determine whether p-p65 was involved in DNCB-induced AD-like skin lesions revealed that the p-p65 expression was significantly increased in the DNCB group compared to the control (Fig. 2C). However, the AYZ treatment significantly decreased the p-p65 expression. Thus, the immunohistochemical analysis of p-p65 expression was consistent with the findings in the Western blot analysis. Besides, a significant increase in IL-1β, TNF-α was observed in the DNCB group compared to the control group (*p* < 0.01; Fig. 2D-E). In contrast, the TNF-α, IL-1β expressions were significantly decreased in the AYZ group compared to the DNCB group (*p* < 0.01).

### 3.3 AYZ inhibited the activation of NLRP3 inflammasome in the skin tissues

**Fig. 3:**
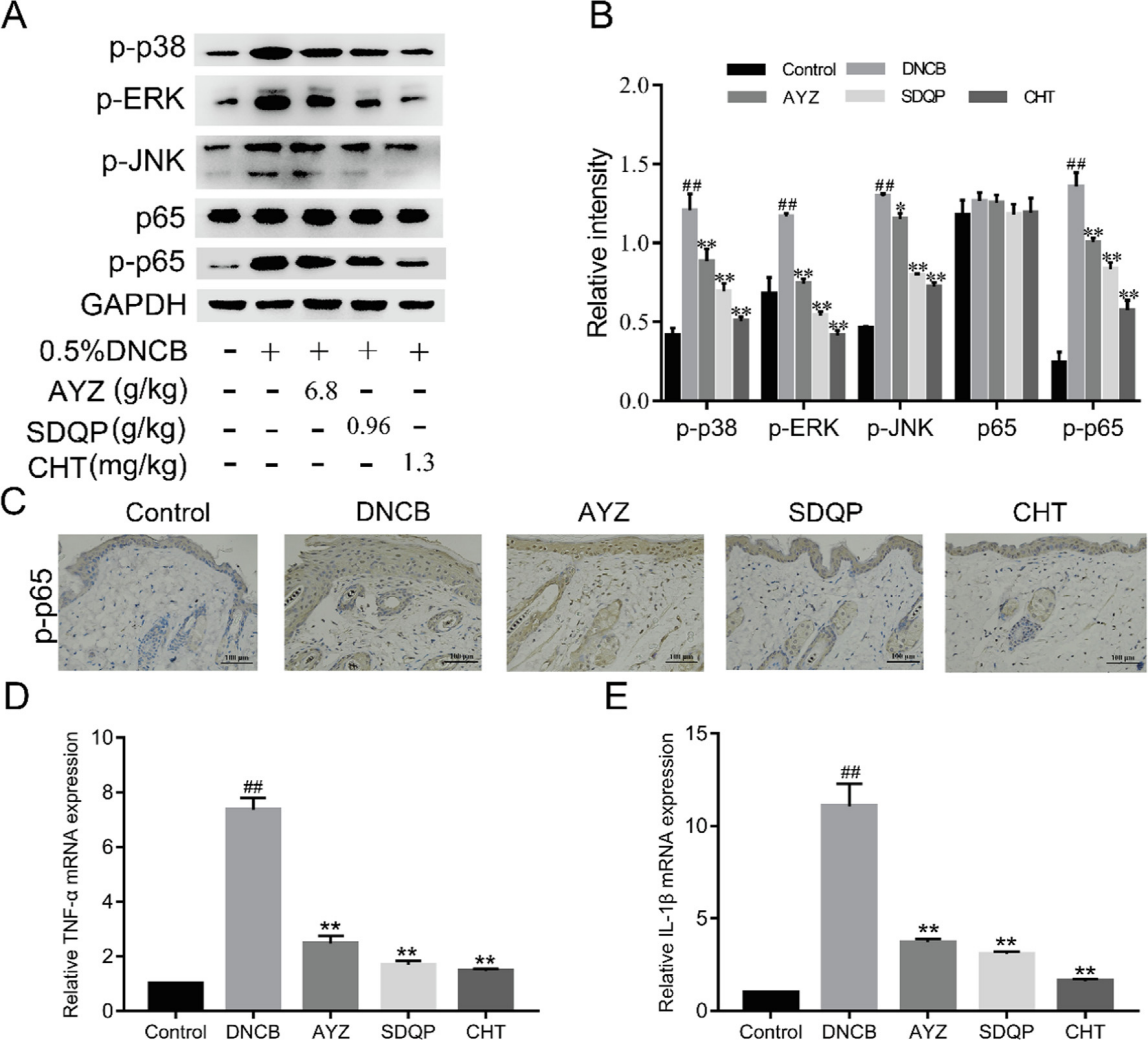
Angelica Yinzi suppresses the production of inflammatory cytokines via the MAPKs/NF-κB pathway. (A-B) The Western blot analysis of p-p38, p-ERK, p-JNK, p65, and p-p65 proteins expressions. (C) The p-p65 expression in the skin tissue based on the immunohistochemical staining at magnification × 200. (D-E) The mRNA levels of TNF-α and IL-1β in each group. Data are expressed as the mean ± SD. (n = 3). ^##^*p* < 0.01, vs. the control group, *p < 0.05 and **p < 0.01 vs. the DNCB group.

The IHC analysis revealed that the stains in mice skin stained with NLRP3, ASC, Caspase-1 antibodies were more intense in the DNCB group compared to the control group (Fig. 3A). However, NLRP3, ASC, and Caspase-1 contents in the skin were significantly decreased following AYZ treatment. In addition, the IL-1β expression in the DNCB group was significantly up-regulated compared to the control group (*p* < 0.01), but the AYZ treatment significantly alleviated this increase (*p* < 0.05; Fig. 3B and C).

### 3.4 The AYZ composition analysis

**Fig. 4:**
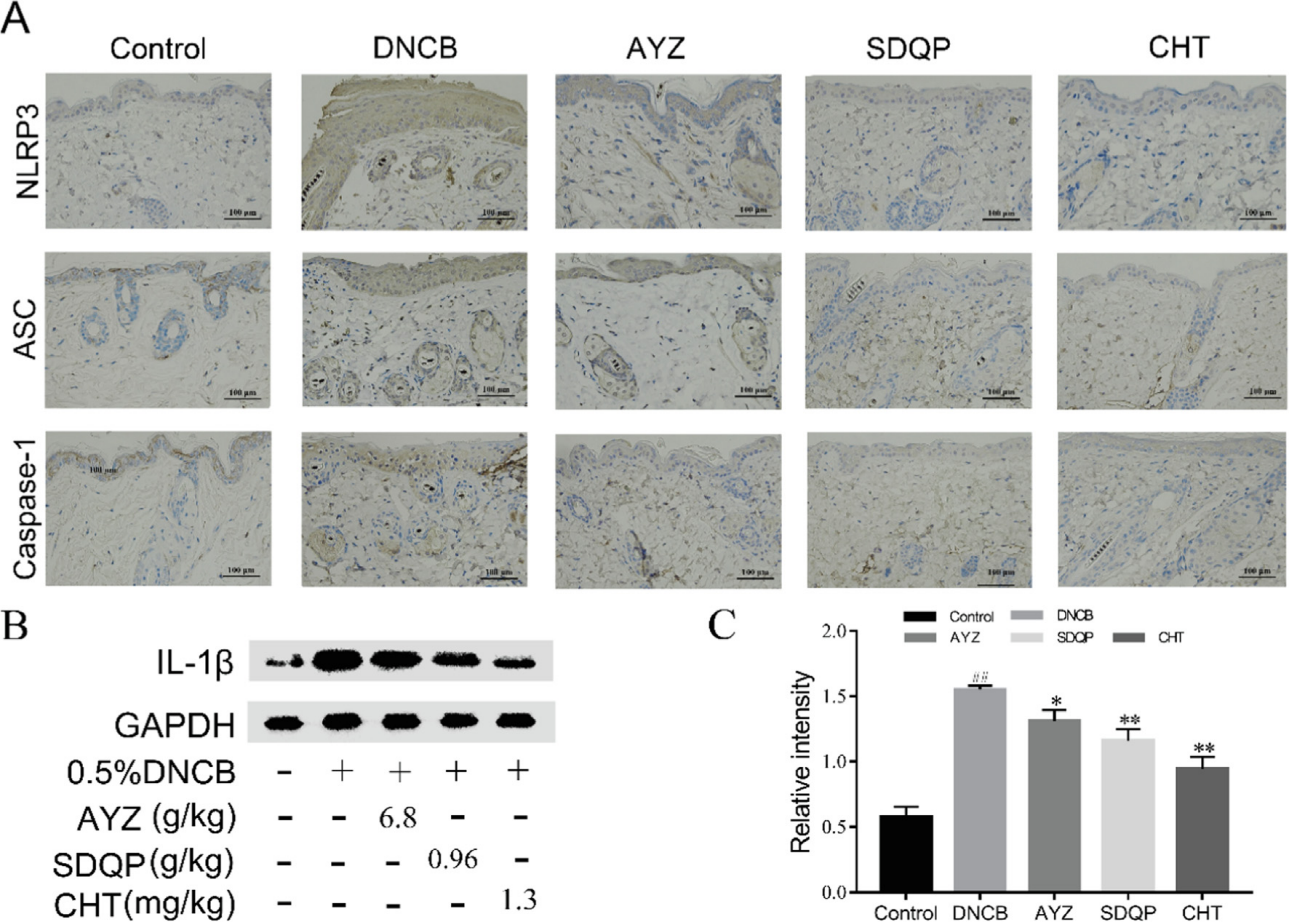
Angelica Yinzi suppresses the NLRP3 inflammasome activation. (A) The NLRP3, ASC, and Caspase-1 expression in the skin tissue based on the immunohistochemical staining at magnification × 200. (B-C) The expression IL-1β proteins were determined by Western blot analysis, and gray values were calculated. Data are expressed as the mean ± SD. (n = 3). ^##^*p* < 0.01, vs. the control group. *p < 0.05 and **p < 0.01 vs. the DNCB group.

**Fig. 5:**
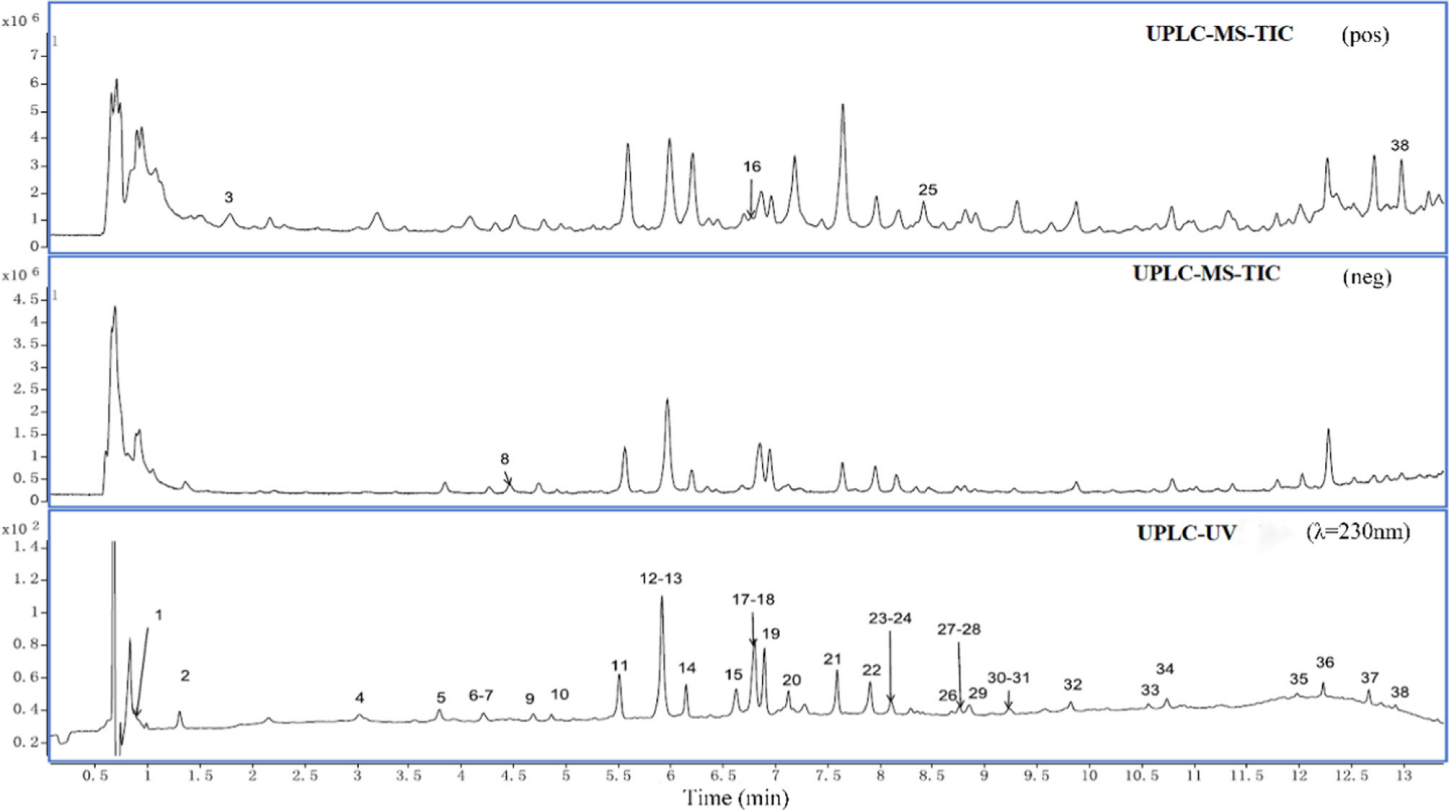
UPLC-UV and UPLC-TOF-MS total ion flow chromatography of Angelica reference solution.

The material standard of AYZ is 11 medicinal flavors. A comparative analysis of the material standard and liquid quality for each component and in a drug formulation revealed the source of the drug flavor by characterizing the main chromatographic peaks. In addition, the UPLC-UV and UPLC-TOF-MS total ion flow chromatography of Angelica reference solution (positive and negative modes) was performed, identifying chromatographic peaks of 1–38, which have been separated and tested well (Fig. 4, Table 1).(See Fig. 5.).

**Table 1:** Compounds present in the Angelica Yinzi formula.

NO.	Compound name	tR (min)	[M + H]+	[M−H]-	Formula	Fragment ions (*m*/*z*)	Resourse
1	Citric Acid	0.913	NA	191.0197	C6H8O7	(-)191.0174,111.0085,87.0088, 59.0121	Pl
2	Gallic acid	1.358	NA	169.0142	C7H6O5	(-)169.0148,125.0241,97.0298, 79.0191	Pl
3	Phenylalanine	1.805	166.0862	NA	C9H11NO2	(+)166.0859, 120.0805, 103.0538	Tt, Lcx, As, Rg, St, Zo
4	Tryptophan	3.080	205.0973	203.0826	C11H12N2O2	(+)205.0975,188.0697,170.0595,146.0597, 118.0652	Tt, Lcx, As, St
5	P-hydroxybenzylmalnic acid	3.841	NA	209.0455	C10H10O5	(-)209.0465, 165.0553, 121.0659, 93.0349, 59.0149	Gu
6	Oxypaeoniflorin	4.237	519.1477	495.1512	C23H28O12	(-)495.1525, 137.0246	Pl
7	Catechin	4.239	291.0862	289.0718	C15H14O6	(-)289.0720, 245.0812,125.0241	Pl
8	2-propylbutanedioic acid	4.465	161.0808	159.0663	C7H12O4	(-)159.0657,141.0543,115.0763,97.0661	St
9	Vanillic acid	4.748	169.0495	167.0350	C8H8O4	(-)167.0348, 123.0450, 79.0558	As, Lcx
10	Neoisoliquiritin	4.910	419.1338	417.1190	C21H22O9	(-)417.1205, 255.0665 135.0086	Gu
11	Albiflorin	5.576	481.1704	525.1614	C23H28O11	(-)479.1569 121.0296	Pl
12	Paeoniflorin	5.942	503.1524	525.1614	C23H28O11	(-)525.1625,449.1456,327.1083,121.0294	Pl
13	2,3,5,4′tetrahydroxystilbene-2-o-β-D- glucoside	6.017	429.1156	405.1193	C20H22O9	(-)405.1191,243.0659	Pm
14	Prim-O- glucosylcimifugin	6.221	469.1704	513.1613	C22H28O11	(-)513.1622,467.1562,305.1022, 161.0456	Sd
15	Ferulic acid	6.670	195.0651	193.0506	C10H10O4	(-)193.0501,178.0269,160.8418,149.0605,134.0372	Lcx, As
16	Pistil oflavone-7-o-β-D-glucoside	6.749	447.1288	491.1196	C22H22O10	(-)491.1199,283.0614	Am
17	Liquiritin apioside	6.807	573.1578	549.1621	C26H30O13	(-)549.1609,417.1173,255.0661,135.0087	Gu
18	Liquiritin	6.861	441.1156	417.1190	C21H22O9	(-)417.1184,255.0659,135.0090	Gu
19	Trans tilbene glycoside*	6.965	407.1338	405.1190	C20H22O9	(-)405.1199, 243.0662	Pm
20	Cimifugin	7.179	307.1176	NA	C16H18O6	(+)307.1181,289.1073,259.0599,235.0600	Sd
21	4-O-β-Dglucosyl-						
5- Omethylvisamminol	7.614	453.1757	497.1665	C22H28O10	(-)497.1659,451.1622,271.0987	Sd	
22	Hesperidin	7.913	611.1971	609.1829	C28H34O15	(-)609.1818, 301.0718	St, Tt
23	Mudanpioside I	8.192	503.1524	525.1618	C23H28O11	(-)525.1610,479.1557,121.0289	Pl
24	Rosmarinic acid	8.194	NA	8.194	C18H16O8	(-)359.0775,197.0452,161.0242	St
25	Senkyunolide I	8.402	247.0942	NA	C12H16O4	(+)247.0937, 207.1010	Lcx, As
26	Isoliquiritin	8.720	419.1335	417.1192	C21H22O9	(-)417.1179,255.0652,135.0079	Gu
27	Ononin	8.787	431.1336	475.1246	C22H22O9	(-)475.1236,267.0666	Gu, Am
28	Rabdosiin	8.820	NA	717.1462	C36H30O16	(-)717.1453,519.0932,321.0403	St
29	Glychionide A	8.895	447.0925	445.0774	C21H18O11	(-)445.0781,269.0459,113.0245	Gu
30	5-Omethylvisamminol	9.288	291.1226	NA	C16H18O5	291.1226,243.0647,219.0648,191.0696	Sd
31	Liquiritigenin	9.292	257.0808	255.0661	C15H12O4	(-)255.0663, 135.0088, 119.0504	Gu
32	*sec*-o- glucosylhamaudol	9.852	439.1599	483.1509	C21H26O10	(-)483.1517,437.1463,257.0821,179.0554	Sd
33	5R-3-Heptanone,5-						
hydroxy-1,7-bis	10.634	397.1621	373.1661	C21H26O6	(-)373.1656,179.0716	Zo	
34	Benzoylpaeoniflorin	10.786	602.2227	629.1876	C30H32O12	(-)629.1876,553.1706,121.0293	Pl
35	Licorice saponin G2	12.031	839.4059	837.3914	C42H62O17	(-)837.3923,351.0584,193.0344	Gu
36	glycyrrhizic acid	12.314	845.3933	821.3964	C42H62O16	(-)821.3931, 351.0557	Gu
37	6-Gingero	12.724	294.1825	NA	C17H26O4	NA	Zo
38	Senkyunolide A	12.978	193.1221	NA	C12H16O2	193.1215,175.1110,147.1163,137.0593	Lcx

Compared with the standard compound. As: Angelica sinensis (Oliv.) Diels; Pl: Paeonia lactiflora Pall.; Lcx: Ligusticum chuanxiong Hort.; Rg: Rehmannia glutinosa Libosch.; Tt: Tribulus terrestris L.; Sd: Saposhnikovia divaricata(Trucz.) Schischk.; St: Schizonepeta tenuisfolia Briq.; Pm: Polygonum multiflorum Thunb.; Am: Astragalus membranaceus (Fisch.) Bge.; Gu: Glycyrrhiza uralensis Fisch.; Zo: Zingiber officinale Rosc.

## 4 Discussion

Atopic dermatitis is a complicated chronic inflammatory disease caused by the interaction of genetic factors through the stimulation, triggering IgE-mediated forms of skin inflammation and allergic reaction (Xiong et al., 2021). In the present study, AYZ effectively alleviated DNCB-induced AD-Like symptoms and reduced AD-induced inflammation by suppressing the activation of the NLRP3 inflammasome and the MAPKs/NF-kB signaling pathway.

Traditional herbal extracts are widely used in Asia as folk remedies for inflammatory diseases, including AYZ (Choi et al., 2011). Therefore, this study identified and characterized the compounds present in AYZ UPLC-TOF-MS. Angelica Yinzi contains 38 chemical components, including citric acid, gallic acid, ferulic acid, and cimifuginin (Table 1). Citric acid alleviates DNCB-induced AD in animals (Inoue et al., 2010), while gallic acid contributes to the *in vivo* anti-inflammatory activities of PHF against AD, where it exert its anti-atopic and anti-inflammatory activity on the skin and in the immune system (Tsang et al., 2016). Ferulic acid also alleviated AD-like symptoms in mice through its potent anti-inflammatory effect (Zhou et al., 2020). Besides, cimifugin suppresses allergic inflammation by reducing the epithelial-derived initiative key factors by regulating the tight junctions (Wang et al., 2017). Therefore, AYZ possibly alleviated DNCB-induced AD because it contains these compounds.

Repeated allergic inflammatory reactions cause skin surface remodeling and hardening, epidermal thickening, and rupture, which are histological characteristics of AD, due to the mast cells and macrophages infiltration into the skin tissue (Fujii et al., 2009, Modena et al., 2016). The histamine released by activated mast cells induces skin itching, dryness, scab, and bleeding, which are AD indicators Xiong et al., 2021). In the present study, the dermatitis score, thickening with scabbing, hemorrhage, and edema in the dorsal skin were significantly reduced in the AYZ group. Besides, H&E and TB staining revealed that the mice skin thickening and inflammatory cells infiltration were significantly relieved in the AYZ group. At the same time, AYZ markedly down-regulated the IgE levels in the serum. The IgE hypersecretion is the primary AD etiology (Park et al., 2021). IgE binds to high-affinity for IgE-Fc receptor type I on the surface of mast cells, releasing various types of inflammatory mediators (Dupuy, 1994, Werfel et al., 2016). Overall, AYZ alleviated AD symptoms by repressing IgE accumulation and infiltration of mast cells in the skin.

Moreover, MAPKs and NF-κB signaling pathways are closely associated with AD(Sur et al., 2019). The MAPK signaling modules are divided into three groups, including ERK, JNK, and p38 (Duan and Wong, 2006; Huang et al., 2019), which increase intracellular pro-inflammatory cytokines and responses through inflammatory responses in various immune cells. These signaling modules were significantly downregulated by AYZ in this study.

Besides, NF-κB is an essential downstream target of MAPK signaling, which regulates many inflammatory cytokines, including TNF-α and IL-1β (Fann et al., 2018, Zhao et al., 2019). TNF-α and IL-1β drive the inflammatory cascade, activating innate immunity and subsequent inflammatory responses (Koga et al., 2008) and repressing the TNF-α and IL-1β expressions positively influence AD (Chen et al., 2020). Inflammation, as an important response to infection in the organism, is of great importance for the development of disease. It has been shown that inhibition of p38 (Nadeem et al., 2017), JNK (Chamcheu et al., 2019), ERK (Chen et al., 2021) and NF-κB (Nadeem,Ahmad, 2017) signaling pathways can alleviate other chronic inflammatory skin diseases including psoriasis(Nadeem et al., 2015). This is consistent with the results of the present study, where AYZ blocked ERK, JNK, p38 phosphorylation and NF-κB signaling pathways, while inhibiting TNF-α and IL-1β expression in the DNCB group.

The NF-κB signaling pathway is a critical pathway triggering the NLRP3 transcription (Li et al., 2021). It is commonly phosphorylated at the Ser536 position, then translocated to the nucleus up-regulating the NLRP3 and IL-1β mRNA expressions (Wu et al., 2017). The NLRP3 interacts with the ASC via a thermal protein domain leading to the cleaving of pro-caspase-1 into mature caspase-1, which, when activated, converts IL-1β into mature IL-1β with proinflammatory functions, subsequently leading to cell death (Zhao et al., 2019). Besides, acne induces inflammation by activating the NLRP3 inflammasome through the MAPK/NF-κB signaling pathway (Fang et al., 2020). Thus, the decreased activated NLRP3, Caspase-1and ASC induced by DNCB implies that the AYZ treatment markedly repressed the activation of the NF-κB pathway and NLRP3 inflammasome. Nevertheless, there were some limitations in this study. At present, only 38 compounds have been identified. However, it is impossible to determine which compounds exert an effect on AD.

## 5 Conclusions

We found that AYZ decreased serum IgE levels, reduced the epidermal thickness of AD-like lesioned skin, and inhibited the infiltration of mast cells into AD-like lesioned skin. AYZ also reduced the expression of pro-inflammatory factors TNF-α and IL-1β by inhibiting MAPKs/NF-kB signaling pathway. In addition, we demonstrated that NLRP3 inflammasome activation was significantly downregulated after AYZ treatment. In conclusion, AYZ ameliorated the symptoms of AD. AYZ inhibited the proliferation of mast cells, suppressed the activation of NLRP3 inflammasome and MAPKs/NF-kB signaling pathway, reduced the infiltration of inflammatory cells, and ameliorated DNCB-induced AD-like skin inflammation in mice. These findings provide a theoretical basis for the potential application of AYZ in AD treatment.

## 6 Financial support

This work was supported by the Hubei Provincial Natural Science Foundation of China (No.2018CFB657).

## CRediT authorship contribution statement

**Wei Liu:** Conceptualization, Methodology, Software. **Wanci Song:** Methodology, Data curation, Writing – original draft. **Yang Luo:** Visualization, Investigation. **Hanxiong Dan:** Supervision. **Li Li:** Software, Validation. **Zhouyang Zhang:** Software, Validation. **Daonian Zhou:** Supervision. **Pengtao You:** Writing – review & editing.

## Declaration of Competing Interest

The authors declare that they have no known competing financial interests or personal relationships that could have appeared to influence the work reported in this paper.

## Data Availability

The data in this study is available from the corresponding author upon reasonable request.
